# Evaluation of Salivary *Streptococcus mutans* and Dental Caries in Children with Heart Diseases

**DOI:** 10.15171/joddd.2015.021

**Published:** 2015-06-10

**Authors:** Behjatolmolook Ajami, Ghazale Abolfathi, Eftekhar Mahmoudi, Zahra Mohammadzadeh

**Affiliations:** ^1^Professor, Department of Pedodontics, Faculty of Dentistry, Mashhad University of Medical Sciences, Mashhad, Iran; ^2^Pedodontist, Private Practice, Mashhad, Iran; ^3^Associate Professor, Department of Pediatric Cardiology, Mashhad University of Medical Sciences, Mashhad, Iran; ^4^Assistant Professor, Department of Pedodontics, Faculty of Dentistry, Mashhad University of Medical Sciences, Mashhad, Iran

**Keywords:** Dental caries, heart diseases, Streptococcus mutans

## Abstract

***Background and aims***. In the presence of certain systemic diseases, oral microflora may aggravate the condition of the disease. Microbial population in the oral cavity especially with heart disease can increase the risk of bacterial endocarditis. The aim of this study was to evaluate the rate of oral *Streptococcus mutansand* the rate of caries in children suffering from heart disease.

***Materials and methods.*** In this cross-sectional research, 66 children with congenital or acquired heart disease and 50 healthy children were selected. Children were orally examined and decayed, missing, and filled teeth (DMFT) index was recorded for each subject. Saliva samples were taken from all subjects, and cultured on a special laboratory media and another specific media for *S. mutans* (sorbitoll +manitol). Bacterial counts were recorded, and for statistical analysis, chi square, Pearson’s, and Exact Fisher tests were performed using SPSS 16 software.

***Results.*** The rate of *S. mutans* in children with congenital heart disease was significantly higher than the rates in childrenwith acquired heart disease and healthy control subjects. The mean DMFT in children with acquired heart disease who tookpenicillin as prophylaxis monthly was significantly lower than the other groups.

***Conclusion***. The results revealed lower oral bacteria counts and comparatively lower caries rates in children with heart diseases, probably because of an effect of the regular prophylactic antibiotic regimen.

## Introduction

Dental caries is one of the most common bacterial diseases and its association with the systemic diseases such as heart disease can cause severe side effects in patients, as infectious foci in the oral cavity especially among children can increase the risk of endocarditis.^1-5^

Congenital heart disease is divided into two major categories including cyanotic (right to left shunt) and acyanotic (left to right shunt). The acyanotic type is associated with complications like ventricular septal defect and atrial septal defect, while obstructive lesions such as pulmonary valve stenosis are the most common cyanotic lesions.^6-8^

Acquired heart disease is usually the consequence of acute rheumatic fever, and patients who take penicillin for a long time usually recover. Fewer number of patients are affected by mitral valve stenosis, which is often the consequence of repeated onset of the disease.^1^

Components of the oral cavity including teeth, tongue, and the oral mucosa are locations for certain types of microbes.^9^ Among oral cavity bacteria are Gram-positive cocci including Streptococci and Staphylococci; and gram-negative cocci including Neisseria and Veillonella. Gram-positive bacillus like lactobacillus and *Corynebacterium diphtheriae*, certain types of fungi and viruses are also found in the oral cavity.^10-14^ In most individuals, Streptococci are common natural flora of the mouth.^2-4,15-17^*Streptococcus mutans* is a main pathogen in dental caries that has also been observed in human heart valves and was isolated from the blood in patients with endocarditis.^3-5,16-17^

Several factors such as the host factors and the diet affect the composition of the oral flora. Antibiotics also have an influence on the oral flora and suppress certain categories of microbes; for example, penicillin eliminates oral bacteria and broad-spectrum antibiotics reduce Gram-positive and Gram-negative bacteria, and thus, provide a suitable environment for fungi and yeast to grow.^18^ Factors that affect the healthy microbial balance in the oral tissues include failure to maintain a favorable oral hygiene, dental plaque accumulation of fermentable diet carbohydrates, systemic diseases that increase the risk of caries, periodontal disease, and various oral lesions.^19^

Children who are affected by heart disease have higher metabolic needs and gain insufficient energy so they need frequent meals.^20^ In addition, salivary excretion in cardiac patients is lower than normal.^21-22^ As a result, cardiac condition may be capable of affecting oral health and bacterial content. The decayed, missing, and filled teeth (DMFT) index has been shown to be higher than normal in heart patients.^17^ Oral *S. mutans* count is also higher in heart patients, and evidence shows this species can cause bacteremia and infectious endocarditis.^20,23,24^

As research on oral bacteria in children with heart disease is scarce, the current study was conducted for evaluating oral bacteria and caries rate in children with congenital heart disease, rheumatic (acquired) heart disease, and healthy children.

## Materials and Methods

This cross-sectional study was conducted on 116 children 3 to 12 years old who referred to a private heart clinic in Mashhad, Northeastern Iran. Study subjects were 50 children with congenital heart disease, 16 patients with acquired heart disease, and 50 healthy children as control group. Because of antibiotic therapy, the prevalence of acquired heart disease has significantly decreased in recent years, and a higher sample for the acquired heart disease group was not available.

A questionnaire was filled for each participant containing demographic data like age, gender, type of heart disease, and oral hygiene by the parents.

For assessing dental caries of participants, the DMFT/dmft index was used. Examination was performed by observation. According to a previous study, there is no difference between visual tactile method and visual method in assessing dental caries. Also the use of an explorer for assessing dental caries is not recommended due to probability of destruction of enamel.^16^

Patient’s saliva was collected using a sterile swab from the lingual area of the mandibular teeth in 15 seconds, and then the sample was transferred to a tube containing Nutrient broth. The tubes were maintained at 37°C in an incubator until test time.

Macconkey agar, blood agar medium, and CTA media containing manitol and sorbitol in distilled water were used to culture sterile swab containing saliva into the broth environment that was in an incubator at 37°C for 24 to 48 hours. Then cultured plates were incubated at 37°C for 24-48 hours and then removed to count the bacteria.

Data were described by number, percent, mean and standard deviation. Chi-square and Fisher’s exact tests were used for comparison. Statistics were calculated using SPSS 16 for Windows software. P value less than 0.05 was considered statistically significant.

## Results

The most frequently observed congenital heart disease in this study was ventricular septal defect (VSD). Fisher’s exact test showed *S. mutans* count is significantly higher in patients with congenital heart disease compared with the control group (P = 0.026).

Comparison of the distribution of the *S. mutans* count among groups with VSD and other congenital heart diseases showed that there is no relationship between the type of the heart disease and the existence of *S. mutans*. *S. mutans* count was lower in patients with acquired heart disease; however, according to Fisher’s exact test, the difference was not significant (Table 1).

**Table 1:** Frequency distribution of *Streptococcus mutans* growth in patients with acquired and congenital heart diseases

	**Growth**	**No growth**	**Total**
	**N(%)**	**N(%)**	**N(%)**
**Acquired heart disease**	0 (0.0)	16 (100.0)	16 (100.0)
**Congenital heart disease**	6 (12.0)	44 (88.0)	50 (100.0)
**Total**	6 (9.1)	60 (90.9)	66 (100.0)
	Fisher s Exact test: p-value=0.32		

According to ANOVA, the difference between mean DMFT (permanent teeth) in three groups was significant (P = 0.013). Tukey test showed that in patients with acquired heart disease the mean DMFT was lower than the other two groups (Table 2).

**Table 2:** Mean and standard deviation of DMFT in the studied groups

	**N**	**Mean**	**SD**	**Min**	**Max**	**Test result**
**Acquired heart disease**	16	1.12	1.25	0	4	
**congenital heart disease**	27	2.81	2.74	0	13	F= 4.59 P=0.013
**Control**	28	3.92	3.72	0	15		
	71	2.87	3.10	0	15	

The distribution of salivary bacteria among study groups revealed that bacterial load in patients with acquired heart disease was lower than the other two groups (Table 3).

**Table 3:** Frequency distribution of bacterial growth in saliva samples of studied groups

	**Growth**	**No growth**	**Total**
	**N(%)**	**N(%)**	**N(%)**
**Acquired heart disease**	3 (18.8)	13 (81.3)	16 (100.0)
**congenital heart disease**	32 (64.0)	18 (36.0)	50 (100.0)
**Control**	34 (68.0)	16 (32.0)	50 (100.0)
**Total**	69(59.5)	47 (40.5)	116 (100.0)
	P-Value=0/002		x^2^12.9	

## Discussion

In the present study, VSD was the most common congenital heart disease among the studied population, which is similar to the data in the literature.^1,3^*S. Mutans*, as the main cause of dental caries, was significantly more common in patients with congenital heart disease compared with the healthy control group. This finding is in line with previous studies, including one conducted in the UK on more than one hundred children with congenital heart disease.^17,20,24^

Previous studies have not compared *S. mutans* and other bacteria as the cause of dental caries in VSD and other congenital heart diseases. The current study shows that the type of the congenital heart disease is not correlated with the *S. mutans* accumulation in saliva. In addition, there is no difference in *S. mutans* accumulation in saliva between individuals with VSD and those with other congenital heart diseases. With respect to other bacterial groups including *Streptococcus viridans*, *Staphylococcus aureus*, not-pathogen staphylococci, lactobacilli, and candida, there was no difference among patients with congenital heart disease and healthy controls. In addition, bacterial plates with no growth were significantly more in acquired heart disease patients than other two groups. The reason for this reduced salivary bacterial load could be the regimen of monthly antibiotic prophylaxis prescribed for acquired heart patients. However, further controlled studies are required to confirm this theory.

In the present study, the mean DMFT among acquired heart disease patients was significantly lower than those in the other two groups. The mean DMFT among congenital heart disease patients was 2.81, compared to 3.08 in a previous study in Mashhad,^3^ and 3.8 in another study in the UK.^25^ The discrepancy in the mean DMFT between the studies can be related to factors associated with increased awareness of patients regarding issues like nutrition habits and dental care. The significantly lower mean DMFT of 1.12 in patients with acquired heart disease may be due to monthly prophylactic penicillin injections in these patients. No bacterial growth in the salivary samples was considerably higher in acquired heart disease group (83%) than the other two groups (36% in congenital heart disease and 32% in the control group). High levels of bacteria are associated with high dental plaque levels and increased risk of bacterial endocarditis in patients with congenital heart disease,^26^ and the low bacterial levels and DMFT in patients with acquired heart disease in the present study show the importance of monthly penicillin prophylaxis for their oral health.

The results of this study also showed that there is no difference between the distribution of other bacteria, including *Streptococcus viridans*, *Staphylococcus aureus*, non-pathogen staphylococci, lactobacilli, and Candida fungus among the studied groups.

Considering the findings of the present study, it is recommended that antibiotic prophylaxis be used in patients with congenital heart disease who have a higher risk of caries in certain periods such as early eruption of permanent teeth. Taking measures to further increase the knowledge of oral health among parents of such children is also recommended. Prophylactic antibiotic is also recommended to reduce the risk of infectious endocarditis caused by oral bacteria in patients with a compromised immune system.^8^ Due to the higher frequency of oral *S. mutans* in patients with congenital heart disease, daily use of chlorhexidine varnish and fluoride mouthwash is recommended.
